# JOURNEY TO GUIDE: ONE PRACTICE’S ROAD TO IMPLEMENTATION

**DOI:** 10.1093/geroni/igae098.4010

**Published:** 2024-12-31

**Authors:** Carolyn Clevenger, Laura Medders

**Affiliations:** Emory University, Atlanta, Georgia, United States; Emory University, Atlanta, Georgia, United States

## Abstract

In July 2023, Guiding and Improved Dementia Experience (GUIDE) was announced by the Center for Medicare and Medicaid Innovation Center. GUIDE was proposed to test the efficacy of an alternate payment methodology for participating dementia care models committed to providing an enhanced quality of care. The program is hoping to improve quality of life, reduce total cost of care, reduce inappropriate prescribing and healthcare utilization, and delay nursing home placement. GUIDE will pay for comprehensive clinical services as well as caregiver support services. Emory’s Integrated Memory Care has been providing comprehensive dementia care services in our unique model since 2015. However, the IMC has had to adapt our current practice to meet the new requirements of GUIDE. The IMC will discuss implementing the necessary requirements for the GUIDE program and how we adapted our current clinic model to meet the GUIDE requirements. The IMC has had to establish additional processes for intake—including a home visit, utilize different required assessments and measures, continue providing caregiver support, and respite care.

